# Atypical Case of Lyme Neuroborreliosis With Hyponatremia

**DOI:** 10.7759/cureus.24413

**Published:** 2022-04-23

**Authors:** Dhara Rana, Mujtaba Chohan, Nagwa Hafez

**Affiliations:** 1 Osteopathic Medicine, Rowan University School of Osteopathic Medicine, Stratford, USA; 2 Internal Medicine, St. Joseph's University Medical Center, Paterson, USA

**Keywords:** ceftriaxone, peripheral neuropathy, hyponatremia, neuroborreliosis, lyme disease

## Abstract

Lyme neuroborreliosis is diagnostically challenging because of its diverse manifestations. Literatures studies have documented a neurological spectrum that includes radiculoneuritis, lymphocytic meningitis, and cranial neuropathy in the early disseminated stage of Lyme’s disease. Severe and refractory hyponatremia is a rare association with Lyme neuroborreliosis, further misleading clinicians to misdiagnose the syndrome of inappropriate antidiuretic hormone secretion (SIADH). This case report describes a 58-year-old woman who developed progressive lower extremity weakness and paresthesia, cerebellar ataxia, and persistent hyponatremia. The patient was hospitalized to rule out cerebral vascular stroke, Guillain-Barre, and SIADH. Lyme neuroborreliosis was diagnosed and treated with 2mg ceftriaxone from clinical suspicion. With treatment initiation, the patient’s neurological symptoms of gait instability, hyponatremia, and bilateral lower extremities weakness gradually resolved.

## Introduction

The most common vector-borne disease in the northern hemisphere is Lyme disease, caused by the spirochete bacteria Borrelia Burgdorferi [1-3]. Lyme disease is a multistage and multisystem disease mainly affecting but not limited to the skin, joints, heart, and nervous system. The clinical presentation varies with the disease stage. The neurological manifestation collectively known as Lyme neuroborreliosis is reported in up to 12% of patients with Lyme disease [4]. Severe hyponatremia due to Lyme neuroborreliosis is rare, and only a few case reports have been written in literature [5-9]. However, the varying presentation, especially without the classic “bull’s eye” or erythema migran rash, makes it difficult to diagnose Lyme disease. We present a rare case of a female patient with refractory hyponatremia, lumbar radiculopathy, and cerebellar dysfunction symptoms.

## Case presentation

A 58-year-old female with a past medical history of hypertension and anxiety presented to the emergency department (ED) due to one-day symptoms of dizziness, unsteady gait, slurred speech, and tremors but no fever or flu-like symptoms. Additional history revealed an ER visit a few days ago due to symptoms of lower back pain, for which she was treated with NSAIDs that started six days ago.

Physical examination was only remarkable for dysmetria in the upper extremities bilaterally and did not show any rashes, lesions, or other neurological deficits. The patient denies the use of illicit substances and alcohol. Her outpatient medication consisted of hydrochlorothiazide (25mg PO daily) for her hypertension and escitalopram (Lexapro, 10mg PO daily) to manage her anxiety. 

Initial suspicion for a possible neurologic etiology such as stroke led to an immediate CT scan of the brain, which showed no positive mass effect, infract, hemorrhage, or extra-axial collection, and ventricle and sulci were normal in size. Initial labs include elevated WBC (6x10^3^mm^3^), hyponatremia (116mEq/L), hypokalemia (2.5mEq/L), chloride 68mEq/L, bilirubin 2.2mg/dL, serum osmolality 252mOsm/kg, ALT 27 unit/L and AST 23 unit/L. Further MRA testing of the brain and head to rule out stroke and CVA was done, but results were also negative, with anterior and posterior circulation within normal limits. The patient was admitted to the medical floor due to significant electrolyte abnormalities. 

Multidisciplinary teams consisted of a primary medical team, nephrology, neurology, and critical care team consultation. The initial plan was to slowly begin treating hyponatremia and hypokalemia to avoid osmotic demyelinating syndrome and investigate potential causes. Treatment of electrolyte abnormalities did show slight improvement over the next several days but was still not within normal limits. The patient, however, developed lower extremity weakness and paresthesia during her stay, which raised the suspicion of spinal abnormalities and Guillain-Barre Syndrome. 

**Figure 1:**
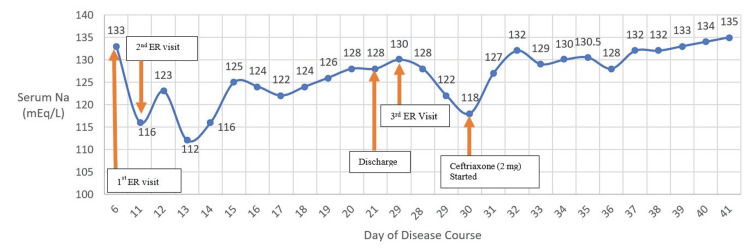
Graph depicts sodium level during the patient’s course at the hospital. When the patient was started on ceftriaxone, her sodium level begin to gradually increase.

An MRI of the lumbar and thoracic spine with and without contrast was performed, which showed degenerative spondylosis at the level of L4/L5. Further, the chest CT scan showed no mediastinal lymphadenopathy or intraparenchymal or pleural mass. It, therefore, ruled out paraneoplastic syndrome, especially since the patient had hypercalcemia (10.2mg/dL) and low back pain. The patient was medically stable on day 12 and discharged to follow up with her primary care physician for metabolic abnormalities (Figure 1). 

After ten days, the patient returned to the ED with worsening bilateral lower extremity weakness, paresthesia, and altered mental status. Given the patient’s persistent serum hypoosmolarity with hyponatremia (Table 1) alongside neurological manifestation, clinical suspicion of a possible diagnosis of CNS infection; more specifically Lyme neuroborreliosis, especially since the patient has symptoms of lower extremity radiculopathy with hyponatremia. 

**Table 1:** Sodium level during the hospital stay

Day of Disease Course	Na serum (mEq/L) (normal 133-145)	Urine Sodium (mEq/L) (normal 15-237)	Urine Osmolarity (mOsm/kg) (normal 50-1200)	Interventions
6	133			1st ed visit and discharged the same day
11	116	37	349	2nd ED visit
12	123	127	357	
13	112	25	298	
14	116	15	418- 476	
15	125	82	484 - 533	
16	124	161	346 - 450	
17	122	45	246 - 321	
18	124	76	450 - 711	
19	126	12-32	724 - 852	
20	128		467	
21	128	129	458	Discharged due to medical stability
29	130			3rd ED visit and Readmission
28	128			
29	122	157	560	ELISA Positive Lyme titers Western blot positive IgM and negative IgG Ceftriaxone 2 mg started
30	118			
31	127	71	544	
32	132			
33	129			
34	130			
35	130.5			Lumbar puncture performed Western blot positive for IgM and negative for IgG
36	128	17	507	
37	132			Discharge to Acute Rehab
38	132			
39	133			
40	134			
41	135			

Lyme titers were performed and were positive: IgM 1.67 (<0.12) and IgG 5.64 (<0.20). Ceftriaxone 2 mg/daily IV was initiated. ELISA testing was done to further screen for Lyme disease, which showed positive results. On Western blot, Lyme was positive for the following IgG antibody: P18, P23, and P41 and positive for the following IgM antibody: P23, P41. The Western blot was thus positive for IgM and negative for IgG. A lumbar puncture was done to investigate Lyme disease as a possible etiology further. Lumbar CSF showed an increased WBC (313, normal <6), lymphocytosis (98, normal <80) (Table 2). Western blot on the CSF was positive for IgM antibodies: 23,39,41 and positive for IgG antibodies: 18, 23, and 41. Thus, the western blot for CSF was interpreted as positive for IgM and negative for IgG. Considering the CSF results and clinical presentation, a diagnosis of Lyme neuroborreliosis was established.

**Table 2:** CSF Cerebrospinal fluid analysis (lumbar puncture on Day 35), which indicated bacterial meningitis CSF: Cerebrospinal fluid

CSF	Value
CSF RBC	0
CSF WBC	313
CSF monocyte	2
CSF lymph	98
CSF protein	453
CSF glucose	58

Ceftriaxone 2mg/daily IV was initiated on 10/2 (Table 1, Figure 1) after the patient was found to have positive Lyme titers. The patient began to show remarkable improvements within two days after ceftriaxone (2mg) was started (Figure 1). She could ambulate more than usual, and her hyponatremia started to resolve steadily. The patient was also given physical therapy and was then discharged to subacute rehabilitation, where the patient continued ceftriaxone for four weeks and received physical therapy.

Shortly after initiating IV ceftriaxone, the patient regained their muscle strength and function and was discharged to subacute rehab for further physical therapy. One year later, the patient states that she is doing very well and denies any neurological deficits and motor or sensory deficits in both upper and lower extremities bilaterally.

## Discussion

Lyme disease is an infectious disease caused by the spirochete borrelia burgdorferi and has the potential to affect multiple organs of the body and is transmitted via the tick bites of Ixodes scapularis and Ixodes pacificus [3]. These vectors are prevalent in Northeastern US, from Maine to Maryland, the north-central state of Wisconsin and Minnesota, and northern California [1,2]. After the inoculation of the spirochete into the human host, different clinical symptoms can present at different stages of the disease. Early infection consists of stage 1, which is localized to the skin with the classic erythema migran, followed by stage 2 (the disseminated infection) within days or weeks, and lastly, stage 3 (persistent infection) within months to years [2]. However, this clinical manifestation is variable. 

The Centers for Disease Control and Prevention recommends a two-step serologic procedure for Lyme disease [10] to diagnose Lyme disease. First, ELISA for antibodies should be conducted. If the ELISA result is negative, Lyme disease is not present in the patient. If the ELISA result is positive or borderline, a Western blot assessment for IgM and IgG antibodies should be done. The IgM Western blot is considered positive if two of three bands (ospC 24, 39, 41) are detected, and the IgG Western blot is considered positive if 5 of 10 bands (18, 23, 28, 30, 39, 41, 45, 58,66, 93) are detected. Our patient had a positive ELISA. For the Western blot, our patient had positive IgM immunoblot and negative for IgG immunoblot because only 3 of 5 spirochetal proteins were tested positive as per the guidelines. However, a minority of patients with early disseminated Lyme disease demonstrate a Western blot of IgM as positive and IgG negative at presentation [10,11]. IgM antibodies are useful only in patients whose illness is shorter than 3 to 6 weeks in duration when such a case occurs. Beyond this point, the isolated IgM antibodies cannot be interpreted as evidence for Borrelia burgdorferi [10].

Furthermore, the CDC website (https://www.cdc.gov/lyme/diagnosistesting/index.html) recently added a supplemental guidance document developed by the Association of Public Health Laboratories (APHL) to aid in reporting language, interpretation, and guidance regarding Lyme disease serologic test results. According to the APHL, if a patient has a positive ELISA and western blot positive for IgM within 30 days of symptom onset and negative for IgG, results are consistent with acute or recent infection with Lyme disease. In our case, our patient is positive for Lyme disease as the IgM immunoblot is positive within 30 days of her symptoms. A small percentage of patients may take 6 to 8 weeks for an IgG Western blot response to develop with reactivity with five or more spirochetal proteins [12].

 Two types of neuroborreliosis exist. The first is the Lyme meningitis in the second stage, which has clinical features of peripheral neuropathy, radiculopathies, facial palsies, and cranial neuritis [4,13]. The second type of Lyme neuroborreliosis occurs in the third stage of Lyme disease. This involves the brain parenchyma causing nonspecific CNS symptoms, which can be presented similarly to brain tumors or multiple sclerosis [4,13].

Our patient presented similar to the second stage of Lyme meningitis. Unlike the classic cases of Lyme disease where the “bull’s eye” rash is first presented before neurological symptoms, in other cases, Lyme meningitis can be the first evidence of Lyme disease occurring without a history of erythema migran or flu-like illness [13]. Our patient presented without the classic erythema migran and flu-like symptoms but only showed neurological symptoms of cerebellar signs (dysmetria, unsteady gait), dizziness, slurred speech, and hyponatremia and hypovolemia. A retrospective study showed that of those diagnosed with Lyme disease, 13% of those people did not have the classic “bulls-eye” rash [14]. Additionally, approximately half of the people who presented without the classic rash were misdiagnosed, and of those who presented with the rash, the rash was initially missed [14]. Thus, Lyme disease diagnosis remains a challenge with or without the classical erythema migran rash. Ultimately, Lyme disease should be screened in patients with unexplained neurological symptoms and SIADH symptoms of persistent hyponatremia and hypoosmolality living in endemic areas.

Lyme neuroborreliosis is considered a mimicker of many better-known disorders. Many case reports published in the literature mistaken Lyme disease for SIADH, Guillain-Barre, stroke, amyotrophic lateral sclerosis (ALS), and other diseases [5-9,15-19]. Similarly, we also had difficulty diagnosing Lyme disease initially. Before considering Lyme disease, our differential diagnosis consisted of cerebrovascular stroke, paraneoplastic syndrome, Guillain-Barre, and SIADH. From looking at lab values in Table 1, the patient had refractory hyponatremia, which could be due to SIADH (Table 1). 

Another potential cause of hyponatremia could have been our patient’s hypertension medication, hydrochlorothiazide. Hydrochlorothiazide is a thiazide diuretic that has a side effect of hyponatremia. Our patient took a small dose of 10 mg orally daily, making it unlikely to have caused severe hyponatremia. Furthermore, the severe hyponatremia resolved when the patient was started on ceftriaxone (2 mg IV). This indicated that the severe hyponatremia was more likely from Lyme neuroborreliosis than the thiazide diuretic she was taking daily.

In our case, an interesting and rare clinical presentation was the severe and refractory hyponatremia with progressively worsening peripheral polyneuritis. The association between hyponatremia and neuroborreliosis is particularly rare, and as far as we know, only a few cases have been reported in clinical literature [5-9]. In these studies, the patients presented with initial signs of hyponatremia and progressively worsening neurological symptoms, and with the initiation of antibiotic therapy, the hyponatremia and neurological symptoms reversed. Our patient also had severe hyponatremia (Table 3). Similarly, after the administration of ceftriaxone, our patient’s serum sodium begins to rise (Figure 1).

**Table 3:** Case reports and our patient reporting hyponatremia due to Lyme disease

Paper	Patient	Serum Sodium
Da Porto et al. (2019) [5]	62 F	123 mEq/L
Perkins et al. (2006) [6]	73 F	118 mmol/L
Shamim et al. (2011) [7]	64 M	125 mEq/L
Shamim et al. (2011) [7]	84 M	121 mEq/
Siddiqui et al. (2017) [8]	83 F	123mEq/L
Syed et al. (2015) [9]	79 M	125 mEq/L
Our patient	58 F	116 mEq/L

One mechanism of SIADH-like symptoms induced by Lyme neuroborreliosis may be due to the inflammation cascade induced by CNS infection, such as Lyme disease, which causes an increased release of ADH [8]. Another mechanism seen in some cases is that CNS infection can cause SIADH by the “reset osmostat”. This happens when the brain resets its osmostat to maintain serum sodium at a lower concentration than normal by increasing the release of ADH [20]. Our patient supports this mechanism. As we introduced ceftriaxone, a third-generation cephalosporin antibiotic, it reduced the CNS infection and gradually increased the serum sodium level.

## Conclusions

Lyme neuroborreliosis with severe hyponatremia is a rare disease manifestation. Our case describes the difficulty of diagnosing Lyme disease initially in patients with atypical features such as hyponatremia with nonspecific CNS manifestation. The diagnosis of Lyme neuroborreliosis with severe hyponatremia becomes even more difficult when the classic clinical sign of the erythema migran rash is not present. One way to prevent this is to screen patients who present with hyponatremia similar to SIADH and neurological manifestations of Lyme disease, especially those who live or travel to endemic areas. Therefore, early screening and diagnosis with ELISA and western blotting can prevent potential misdiagnosis and exacerbation of the symptoms.
